# Native Mass Spectrometry of BRD4 Bromodomains Linked to a Long Disordered Region

**DOI:** 10.5702/massspectrometry.A0110

**Published:** 2022-12-28

**Authors:** Nanako Azegami, Rina Taguchi, Noa Suzuki, Yusuke Sakata, Tsuyoshi Konuma, Satoko Akashi

**Affiliations:** 1 Graduate School of Medical Life Science, Yokohama City University, 1–7–29 Suehiro-cho, Tsurumi-ku, Yokohama, Kanagawa 230–0045, Japan; 2 School of Science, Yokohama City University, 1–7–29 Suehiro-cho, Tsurumi-ku, Yokohama, Kanagawa 230–0045, Japan

**Keywords:** native MS, intrinsically disordered region, bromodomain, protein–drug complex

## Abstract

The contribution of disordered regions to protein function and structure is a relatively new field of study and of particular significance as their function has been implicated in some human diseases. Our objective was to analyze various deletion mutants of the bromodomain-containing protein 4 (BRD4) using native mass spectrometry to characterize the gas-phase behavior of the disordered region connected to the folded domain. A protein with a single bromodomain but no long disordered linker displayed a narrow charge distribution at low charge states, suggesting a compact structure. In contrast, proteins containing one or two bromodomains connected to a long disordered region exhibited multimodal charge distributions, suggesting the presence of compact and elongated conformers. In the presence of a pan-BET-bromodomain inhibitor, JQ1, the protein–JQ1 complex ions had relatively small numbers of positive charges, corresponding to compact conformers. In contrast, the ions with extremely high charge states did not form a complex with JQ1. This suggests that all of the JQ1-bound BRD4 proteins in the gas phase are in a compact conformation, including the linker region, while the unbound forms are considerably elongated. Although these are gas-phase phenomena, it is possible that the long disordered linker connected to the bromodomain causes the denaturation of the folded domain, which, in turn, affects its JQ1 recognition.

## INTRODUCTION

The physicochemical and biological properties of structured domains have been extensively characterized. In contrast, until recently, little information was available on the biological significance of the disordered regions in proteins. Over the past few decades, the crucial role of disordered regions in eukaryotic proteins has been recognized, and various studies have investigated both intrinsically disordered proteins (IDPs) and intrinsically disordered regions (IDRs).^1–5)^ To characterize IDPs and IDRs, nuclear magnetic resonance, molecular dynamics (MD) simulations, and small-angle X-ray scattering (SAXS) are recognized as effective tools.^6–8)^ In addition, electrospray ionization (ESI) mass spectrometry (MS) under non-denaturing conditions, also known as native mass spectrometry (native MS), has been utilized.^9–11)^ The charge-state distribution observed in native MS is related to the compactness of the protein structure^12,13)^; folded proteins generally present a narrow charge distribution range in the relatively high *m*/*z* region (*i.e.*, low charge states), whereas disordered proteins exhibit a wide charge distribution range from the low to high *m*/*z* region. It has also been shown that ion mobility mass spectrometry (IM-MS) provides additional structural information on IDPs and IDRs in the gas phase.^14–16)^

In the ESI mass spectra of a completely disordered protein (such as a denatured protein prepared in an acidic solution containing an organic solvent), a wide charge distribution is observed across a broad *m*/*z* range.^17–19)^ Acid-denatured proteins generally exhibit intense ions with a high number of charges in the relatively low *m*/*z* region. When they are subjected to IM-MS in the positive ion mode, the collision cross-section (CCS) of charged ions increases with increasing the charge number. Similar results were obtained for IDPs without a structured domain.^20)^ When proteins with a structured domain that is linked to an IDR—prepared in aqueous solutions at neutral pH—are subjected to ESI-IM-MS, multimodal charge distributions are observed.^16,21,22)^ In our previous study on the *Schizosaccharomyces pombe* Swi5–Sfr1 complex, which has a disordered region consisting of ∼130 amino acid residues at the N-terminus of Sfr1, the main ions observed in native mass spectra were the Swi5–Sfr1 complex in low-charge states; this corresponded to a compact structure in the gas phase, although a SAXS analysis was indicative of an elongated structure in solution.^16)^ The observed CCS value of the low charged ions in the IM-MS analysis was ∼56% of the value calculated for the solution structure characterized by SAXS. These results indicate that the disordered region of the Swi5–Sfr1 complex shrunk in the gas phase and that the charge states of the protein observed in native MS represented compact conformers. There was no inconsistency between the CCS values determined using IM-MS and the charge state of each ion. To date, a variety of IDPs and IDRs have been studied using native MS, including IM-MS, but only a small number of proteins containing both structured and disordered regions have been characterized using this technique.^11,16,22)^ One of the reasons for this is the difficulties in preparing a sample due to the instability of the IDRs.

**Figure 1:**
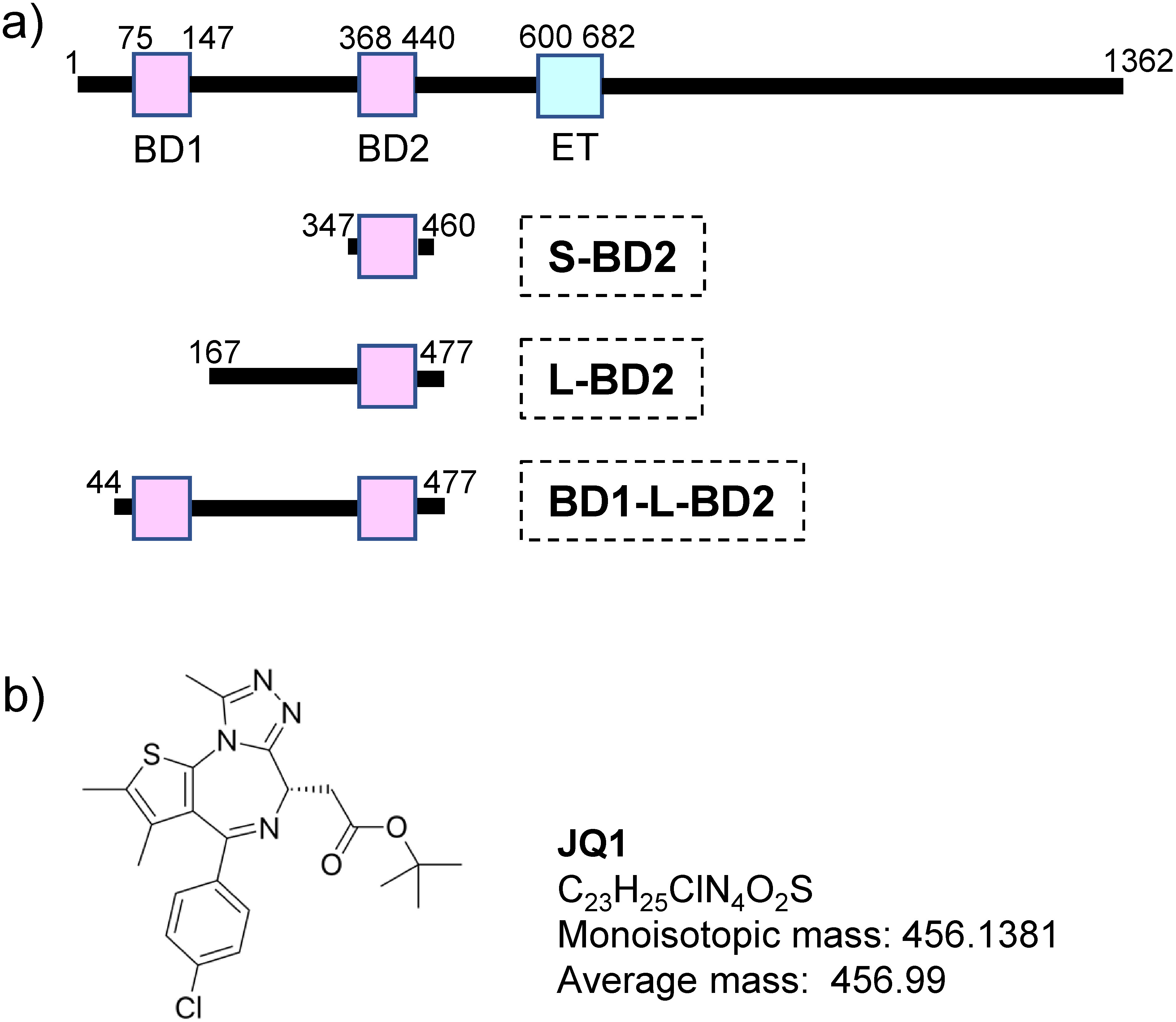
Fig. 1. Domain locations in BRD4 (1-1362) (a) and the chemical structure of JQ1 (b).

In the present study, we characterized three deletion mutants of the bromodomain-containing protein 4, BRD4 (152 kDa), which is a member of the bromodomain and extra-terminal domain (BET) protein family (Fig. 1).^23)^ BRD4 has two tandem bromodomains (BDs), which specifically recognize acetylated lysine in the N-terminal tails of the histone H3 and H4 proteins to recruit transcription factors and coactivators, targeting gene sites to regulate the transcription initiation.^23)^ Compounds that specifically inhibit the binding of acetylated lysine to BDs are generally thought to be potent candidates for therapeutic drugs for the treatment of many human diseases, including cancer and inflammatory disorders.^24)^ The two BDs, BD1 (8.9 kDa) and BD2 (8.6 kDa), which are composed of an evolutionarily conserved four-helical bundle structure, are connected by a long disordered linker comprised of more than 200 amino acid residues (Fig. 1). As demonstrated in several studies using bivalent BD inhibitors,^25–27)^ the simultaneous inhibition of tandem BD in BRD4, in addition to independent BD inhibition, is of great significance for drug development. However, due to the flexibility of the long linker region, no structural information is available for the entire BD1-linker-BD2 region, except for a model structure predicted by the AlphaFold2 program.^28)^

Residues 75–147, 368–440, and 600–682 correspond to the BD1, BD2, and ET domain, respectively. Three protein constructs, S-BD2 (347–460), L-BD2 (167–477), and BD1-L-BD2 (44–477), were prepared and characterized.

In the present study, we analyzed BD2 with a short linker (S-BD2), BD2 with a long linker (L-BD2), and BD1-linker-BD2 (BD1-L-BD2) proteins *via* native MS in the presence and absence of a pan-BET-bromodomain inhibitor, JQ1 (Fig. 1). To compare the BRD4 protein gas-phase and solution structures, we performed size-exclusion chromatography with multi-angle light scattering (SEC-MALS). Based on the results of these experiments, we are now able to discuss the gas-phase behavior of the disordered linker region and bromodomains. The findings reported in this study show that a charge state analysis can provide deep insights into protein conformation, including that of IDRs, even without ion mobility devices. To our knowledge, this is the first report on the gas-phase behavior of a protein that contains two folded domains, with one at either end of a sequence, connected by a long disordered region.

## EXPERIMENTAL

Ammonium acetate (Guaranteed Reagent) was purchased from Nacalai Tesque Inc. (Kyoto, Japan). JQ1 was supplied by ChemScene Inc. (Monmouth Junction, NJ, USA). Dimethyl sulfoxide (DMSO, Guaranteed Reagent) was obtained from Nacalai Tesque and Fujifilm Wako Pure Chemical Corporation (Osaka, Japan). Ultrapure water was prepared using a Puric FP-0120α-UT0 device (Organo Corporation, Tokyo, Japan).

### Preparation of BRD4 proteins

The S-BD2, L-BD2, and BD1-L-BD2 proteins were prepared using an overexpression system in BL21(DE3) strain *Escherichia coli* (*E. coli*).^29)^ The DNA sequence of S-BD2 (residues 347–460) was inserted into a pET-28a vector, which transformed *E. coli* cells. The DNA sequences of L-BD2 (residues 167–477) and BD1-L-BD2 (residues 44–477) were inserted into a pNIC28 vector, used for the transformation of *E. coli*. All bacterial cells were cultured at 37°C in Luria-Bertani (LB) medium containing 50 μg/mL kanamycin. Protein overexpression was induced by adding 0.2 mM isopropyl β-D-thiogalactopyranoside (IPTG, final concentration) to the culture, and cell growth was continued at 15°C for ∼15 h. The cells were harvested by centrifugation (7500×g, 10 min), suspended in lysis buffer (30 mM HEPES (pH 7.4), 400 mM NaCl, 40 mM imidazole, 0.1 mM AEBSF serine protease inhibitor, and 0.1% (v/v) Nonyl Nonidet P-40 non-ionic detergent), and then disrupted by sonication. The supernatant of the crude extract was collected by centrifugation (27000×g, 30 min) and applied to a HisTrap FastFlow column (Cytiva, Tokyo, Japan). His-tagged proteins that were eluted in HEPES buffer (30 mM HEPES (pH 7.4), 400 mM NaCl, 500 mM imidazole, 5% (v/v) glycerol, and 0.1% (v/v) CHAPS surfactant) were further purified by size exclusion chromatography in a HiLoad 26/600 Superdex 200 pg column (Cytiva) (buffer: 30 mM HEPES (pH 7.4) and 400 mM NaCl). After cleavage of the His-tag with thrombin (for S-BD2) or TEV protease (for L-BD2 and BD1-L-BD2), the sample solution was passed through the HisTrap FastFlow column to remove the uncleaved protein fraction (buffer: 30 mM HEPES (pH 7.4), 400 mM NaCl, and 40 mM imidazole). The collected fraction containing resin-unbound proteins was concentrated with an Amicon Ultra 15 centrifugal filter (Millipore, Rahway, NJ, USA), and then subjected to size exclusion chromatography (HiLoad 26/600 Superdex 200 pg, Cytiva) (buffer: 30 mM HEPES (pH 7.4) and 400 mM NaCl). The highly purified proteins were further concentrated using an Amicon Ultra 15, passed through a 0.22 μm filter, frozen in liquid nitrogen, and stored at −80°C until used.

### Sample preparation for nanoESI-MS

The protein solvents for the nanoESI-MS were exchanged with 100 mM ammonium acetate using a BioRad Micro Bio-Spin™ 6 column (Hercules, CA, USA). The pH of the solution was pH 6.8, without adjustment with acids or bases. After solvent exchange, the protein concentration was confirmed by UV absorption data at 280 nm using a DeNovix DS-11 spectrometer (Wilmington, DE, USA). To obtain the mass spectra, 5 μM protein solutions were prepared either with or without 1% DMSO.

A stock solution of 5 mM JQ1 was prepared by dissolving this compound in DMSO. To observe the protein–JQ1 complexes, a protein solution in 100 mM ammonium acetate was mixed with an aliquot of the JQ1 stock solution, resulting in 5 μM protein and 50 or 5 μM JQ1 in 100 mM ammonium acetate containing 1% DMSO.

### NanoESI-MS

Mass spectra were acquired using a SYNAPT G2-HDMS system (Waters, Milford, MA, USA) equipped with a nanoelectrospray ion source in the positive ion mode.^30,31)^ Several microliters of the protein solution were sampled into a nanoESI emitter (HUMANIX, Hiroshima, Japan) or a self-made emitter with an internal diameter of 3–5 μm, and the emitter was set to the nanoelectrospray ion source. The following parameters were used for obtaining mass spectra: 0.65–1.0 kV of capillary voltage, 20 V of sampling cone voltage, 70°C of ion source temperature, 4–20 V of trap collision energy (CE), 1.5–3.0 mL/min of trap Ar gas. The quadrupole profile was set to the automatic mode. The data were processed with the MassLynx v.4.2 software. Molecular masses of the analyte proteins were calculated by the “Manual Find Components” function, which starts by manually picking up two intense peaks in the spectrum.

### Size-exclusion chromatography with multi-angle light scattering (SEC-MALS)

SEC-MALS was conducted using a miniDAWN light scattering detector (Wyatt Technology Corporation, Santa Barbara, CA, USA) downstream of a LC-20AD liquid chromatography system (SHIMADZU, Kyoto, Japan) equipped with a Superdex 200 Increase 10/300 GL gel filtration column (Cytiva). The differential refractive index (SHOKO Science, Yokohama, Japan) downstream of MALS was used to estimate the protein concentration. The running buffer was phosphate-buffered saline (pH 7.4). Twenty microliters of the sample solution containing 0.1 mg of S-BD2, L-BD2, or BD1-L-BD2 were injected into the SEC column, and the protein was eluted at a flow rate of 0.4 mL/min. The data were analyzed using ASTRA version 8.0.1 (Wyatt Technology Corporation).

### Differential scanning fluorimetry—Thermal stability assay

The thermal stability of the BRD4 proteins in solution was assessed using differential scanning fluorimetry (DSF).^32)^ Sample solutions containing 10 μM protein were prepared in 100 mM ammonium acetate or HEPES buffer (30 mM HEPES (pH 7.4) and 400 mM NaCl) and incubated with SYPRO Orange protein gel stain (Thermo Fisher Scientific, Waltham, MA, USA) diluted 1000-fold (w/w). Twenty microliter aliquots of the protein/dye solution were added to a 96-well polymerase chain reaction (PCR) plate, and the emission at 610 nm was measured using a Bio-Rad CFX98 Real-Time System (Hercules, CA, USA), with the temperature being raised from 25°C to 70°C at a rate of 1°C/min. Fluorescence data were normalized to the most intense values in each sample run. The measurements were carried out triplicate for each sample.

## RESULTS AND DISCUSSION

**Figure 2:**
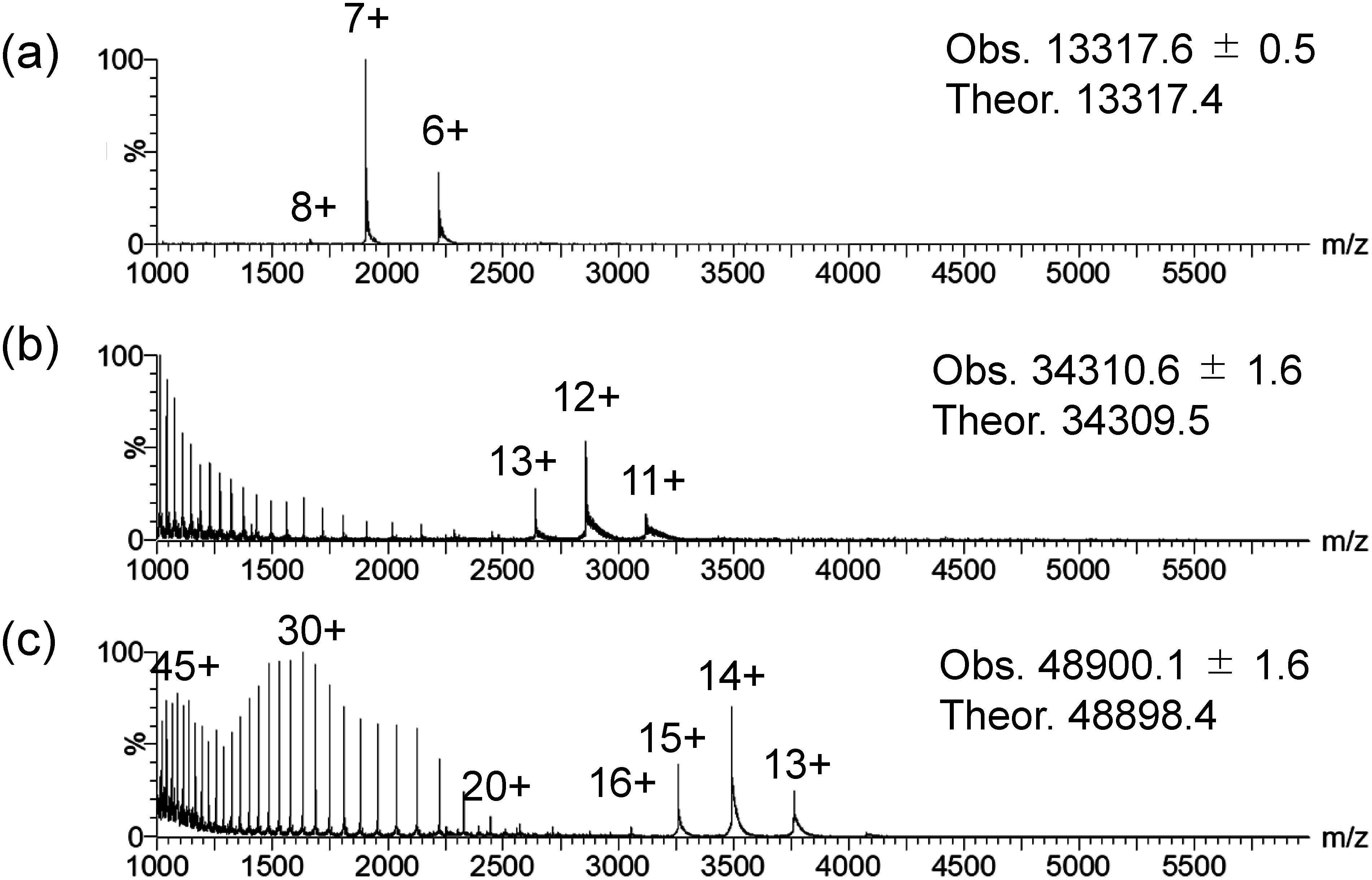
Fig. 2. NanoESI mass spectra of 5 μM (a) S-BD2, (b) L-BD2, and (c) BD1-L-BD2 in 100 mM ammonium acetate.

We first analyzed S-BD2, L-BD2, and BD1-L-BD2 prepared in 100 mM ammonium acetate using nanoESI-MS (Fig. 2). We observed 6+, 7+, and 8+ ions in the mass spectrum of S-BD2, suggesting a compact structure. In contrast, in the mass spectrum of L-BD2, a bimodal charge distribution was observed. L-BD2 exhibited 11+, 12+, and 13+ ions at *m*/*z* 3119.97, 2860.23, and 2640.21, respectively. In addition, highly charged ions were observed at *m*/*z* <2000. Ions with 11+ to 13+ charges correspond to compact conformers, whereas highly charged species correspond to elongated conformers. In the case of BD1-L-BD2, we observed multimodal charge distributions consisting of 13+ to 16+, 19+ to 38+, and >39+ charge states. Ions with charges of 13+ to 16+ correspond to compact conformers, and ions with ≥19+ charges are partially and/or completely unstructured conformers. The linker region of the compact conformers of L-BD2 and BD1-L-BD2 is unlikely to extend in the gas phase. Furthermore, the mass spectrum of BD1-L-BD2 displayed a wider range of charge distribution than that of L-BD2, suggesting that it has more diverse conformers than L-BD2.

Spectra were obtained by applying a trap collision energy of 4 V. The experimentally observed and theoretical masses of the proteins are indicated in each pane.

**Figure 3:**
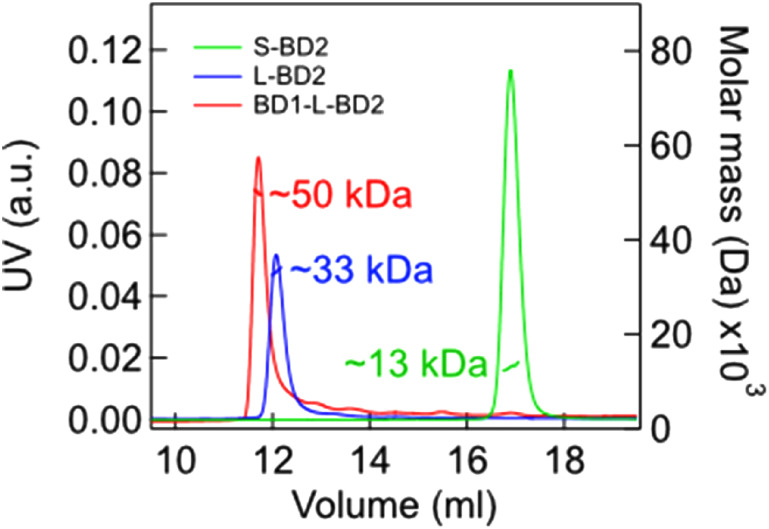
Fig. 3. Elution profiles of S-BD2, L-BD2, and BD1-L-BD2 analyzed by SEC-MALS.

The behavior of the three proteins in solution was then analyzed by SEC-MALS (Fig. 3) to compare their behavior in the gas phase. Our MALS data suggested that the molecular sizes of S-BD2, L-BD2, and BD1-L-BD2 were approximately 13, 33, and 50 kDa, respectively. These figures are in good agreement with the values obtained *via* nanoESI-MS, namely, 13317.6 for S-BD2, 34310.6 for L-BD2, and 48900.1 for BD1-L-BD2. The elution profiles of S-BD2, L-BD2 and BD1-L-BD2 in SEC showed a narrow and nearly normal distribution, suggesting that there was no variation in the molecular shape and radius of gyration. Given that IDRs are generally flexible and elongated in solution, the compact conformers of L-BD2 and BD1-L-BD2, as indicated by low numbers of positive charges in the mass spectra, may have been formed during the extraction of the protein ions from the aqueous solution to the gas phase, that is, ionization, or transfer *in vacuo*.

UV chromatograms at 280 nm of S-BD2, L-BD2, and BD1-L-BD2 (left *y*-axis) are indicated in green, blue, and red, respectively. The experimental molecular sizes of the proteins obtained by MALS (right *y*-axis) are also indicated with lines in the peaks.

Completely disordered proteins, such as α-synuclein and β-casein, exhibit a series of broad charge distributions in mass spectra under non-denaturing conditions.^21,22,33)^ In contrast, proteins that contain both folded regions and IDRs behave differently from completely disordered proteins; the IDRs of these proteins are disordered in the solution phase but compact in the gas phase.^16)^ Such gas-phase behavior has been confirmed by MD simulations, which revealed that the IDRs cling to the folded core region.^16)^ The mass spectra we obtained for L-BD2 and BD1-L-BD2 suggested that a certain percentage of the linker region remained unstructured in the gas phase not only in L-BD2, in which a folded bromodomain is located at one end, but also in BD1-L-BD2, in which a bromodomain is located at each end. These results indicate that the IDRs connected to the bromodomain behave in various ways in the gas phase.

**Figure 4:**
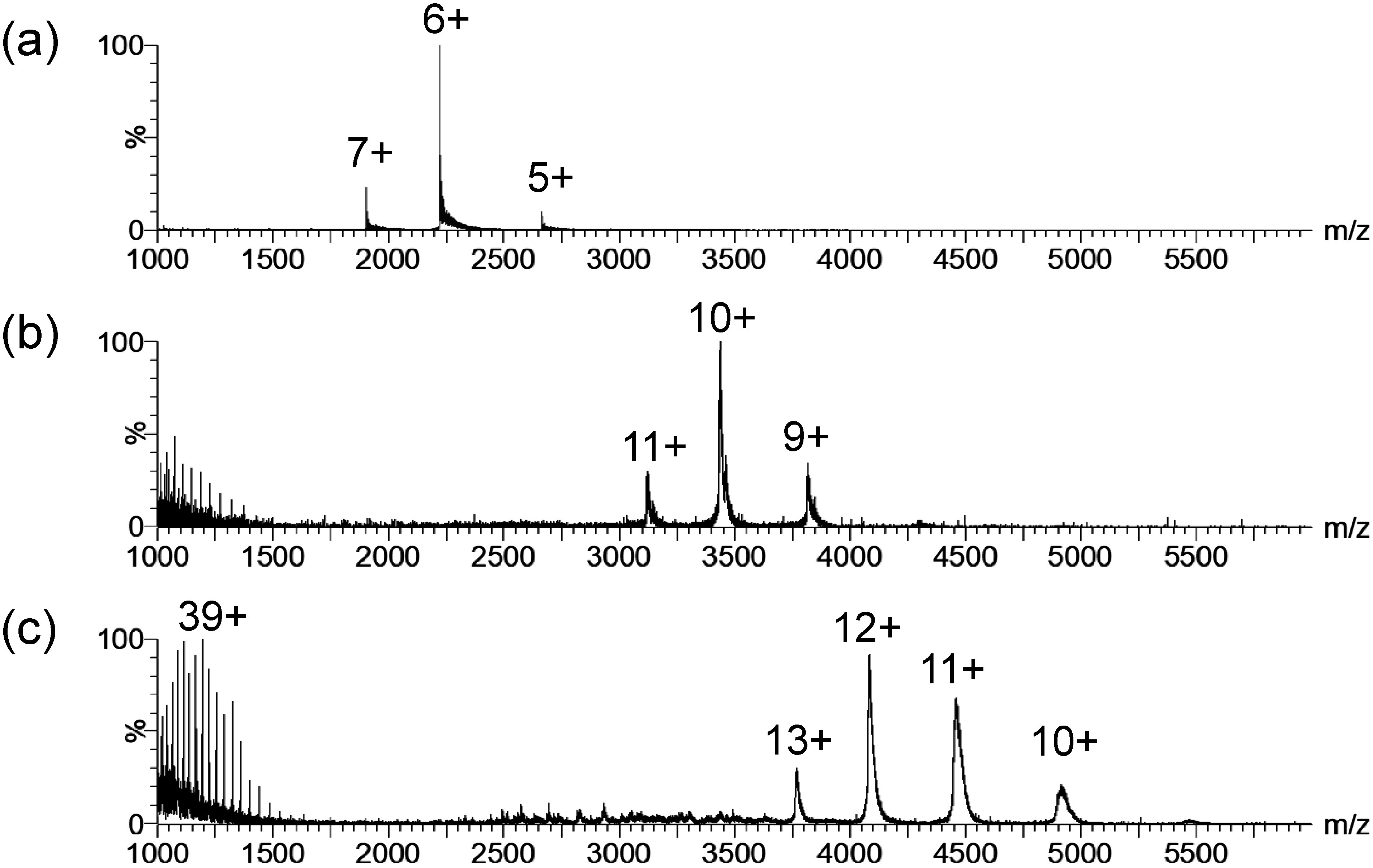
Fig. 4. NanoESI mass spectra of 5 μM (a) S-BD2, (b) L-BD2, and (c) BD1-L-BD2 in 100 mM ammonium acetate containing 1% DMSO.

In subsequent experiments, a pan-BET-bromodomain inhibitor, JQ1, was added to the protein solution and subjected to native MS. Because JQ1 has a low solubility in aqueous solutions at neutral pH, 5 mM JQ1 was prepared in DMSO, and a small amount of the JQ1 solution was added to the protein solution to give a concentration of 5 μM protein and 50 μM JQ1. This working solution contained 1% DMSO, which is the maximum concentration at which protein denaturation can be avoided.^34)^ To analyze the sole effect of 1% DMSO on the nanoESI mass spectra, we subjected 5 μM S-BD2, L-BD2, or BD1-L-BD2 in 100 mM ammonium acetate containing 1% DMSO to nanoESI-MS. As shown in Fig. 4, the addition of 1% DMSO to the sample solutions broadened the L-BD2 and BD1-L-BD2 protein peaks and slightly reduced the protein charge states that were observed in the spectra, consistent with results from a previous study.^34)^ The highest peaks observed for the compact conformers of S-BD2, L-BD2, and BD1-L-BD2 were at 6+, 10+, and 12+, respectively, which were one or two charge states lower than those observed in the absence of DMSO. It is noteworthy that the addition of 1% DMSO also affected the relative ratios of highly and low charged ions in L-BD2 and BD1-L-BD2. In the absence of DMSO, they exhibited a broad charge distribution; however, in the presence of 1% DMSO, the relative population of low-charged ions (*i.e.*, compact conformers) increased, while the relative intensity of the ions at *m*/*z* 1000–2000 decreased and the ions at *m*/*z* 1500–3000 nearly disappeared, resulting in a bimodal distribution. Furthermore, the addition of 1% DMSO caused considerable peak broadening in the L-BD2 and BD1-L-BD2 ions at low-charge states (Figure S2). This effect was not observed for S-BD2, which does not contain a long disordered linker. By applying a CE of 20 V to the trap region behind the quadrupole, the protein peaks were narrowed for the L-BD2 and BD1-L-BD2 samples containing 1% DMSO, as shown in Fig. 4. Given that the peak shape of S-BD2 was negligibly affected by the addition of 1% DMSO, it is possible that the peaks of the compact conformers of L-BD2 and BD1-L-BD2 were broadened by adducts to the IDR.

Spectra were obtained by applying either 4 V (a) or 20 V (b, c) of trap collision energy.

**Figure 5:**
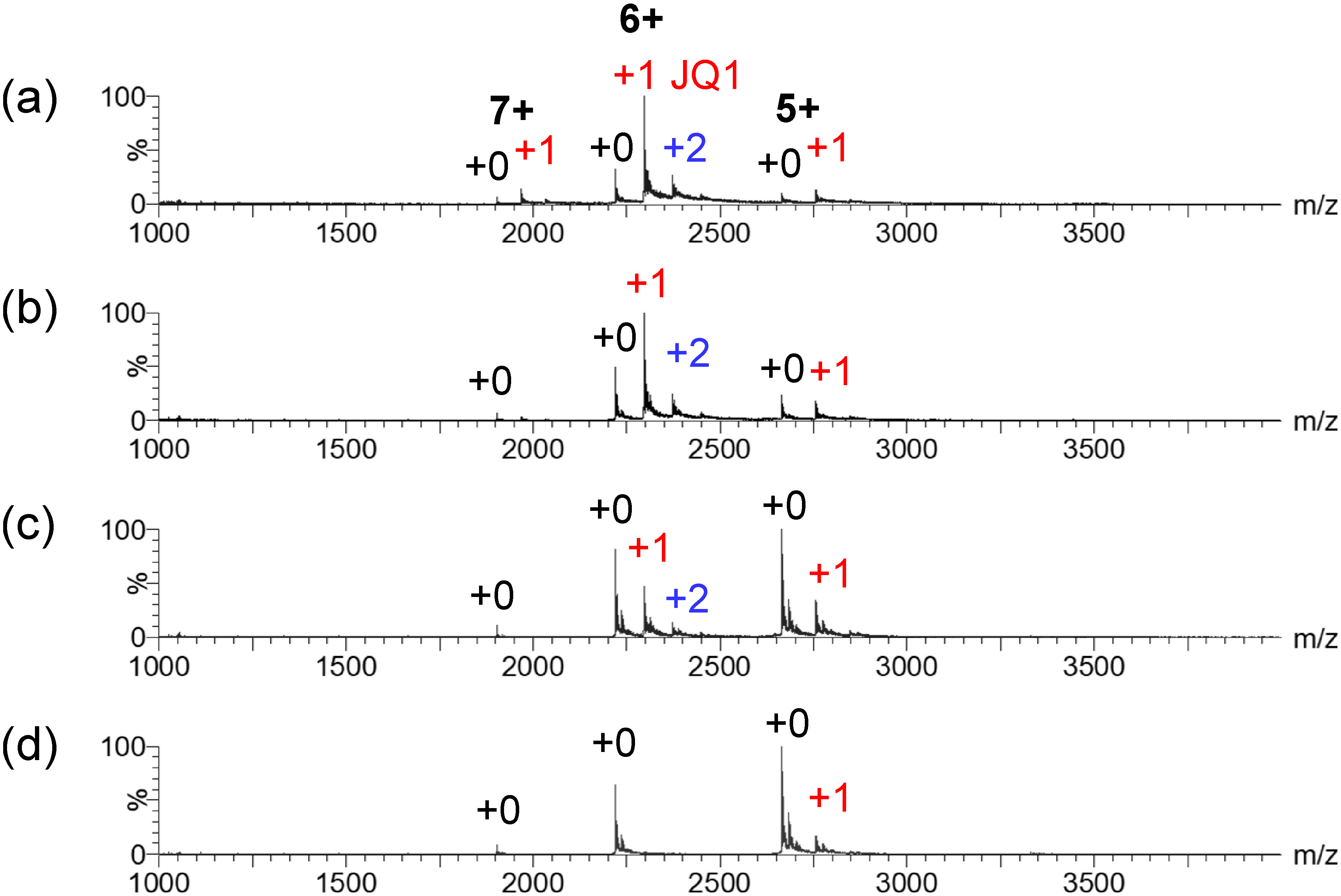
Fig. 5. NanoESI mass spectra of 5 μM S-BD2 with 50 μM JQ1 when applying (a) 4 V, (b) 10 V, (c) 15 V, and (d) 20 V of trap collision energy.

JQ1-binding to each protein was also analyzed using native MS. In the presence of 50 μM JQ1, we observed 5+, 6+, and 7+ charged ions corresponding to a 1 : 1 complex of S-BD2 : JQ1 in addition to the free S-BD2 ions (Fig. 5). Because 10 eq of JQ1 was present in the solution, there were weak peaks corresponding to nonspecific adducts of JQ1 to S-BD2, *i.e.*, the 1 : 2 complex of BD2 : JQ1. In the mass spectrum of 5 μM S-BD2 in the presence of a lower molar concentration of 5 μM JQ1 (Figure S3), the 1 : 2 complex ions disappeared, confirming that they were nonspecific adducts of JQ1 to S-BD2. As shown in Fig. 5, the ions of the 1 : 1 complex were predominantly observed with a narrow peak shape at 4 V CE. When the CE was increased to 20 V, the relative intensities of the complex ions decreased, whereas those of the JQ1-free S-BD2 ions increased. In fact, at 20 V CE, JQ1-free S-BD2 ions are mainly present.

Samples were prepared in 100 mM ammonium acetate containing 1% DMSO. The charge states are indicated in bold letters. The numbers of JQ1 molecules associated with the protein ions are indicated by black, red, and blue letters, respectively.

**Figure 6:**
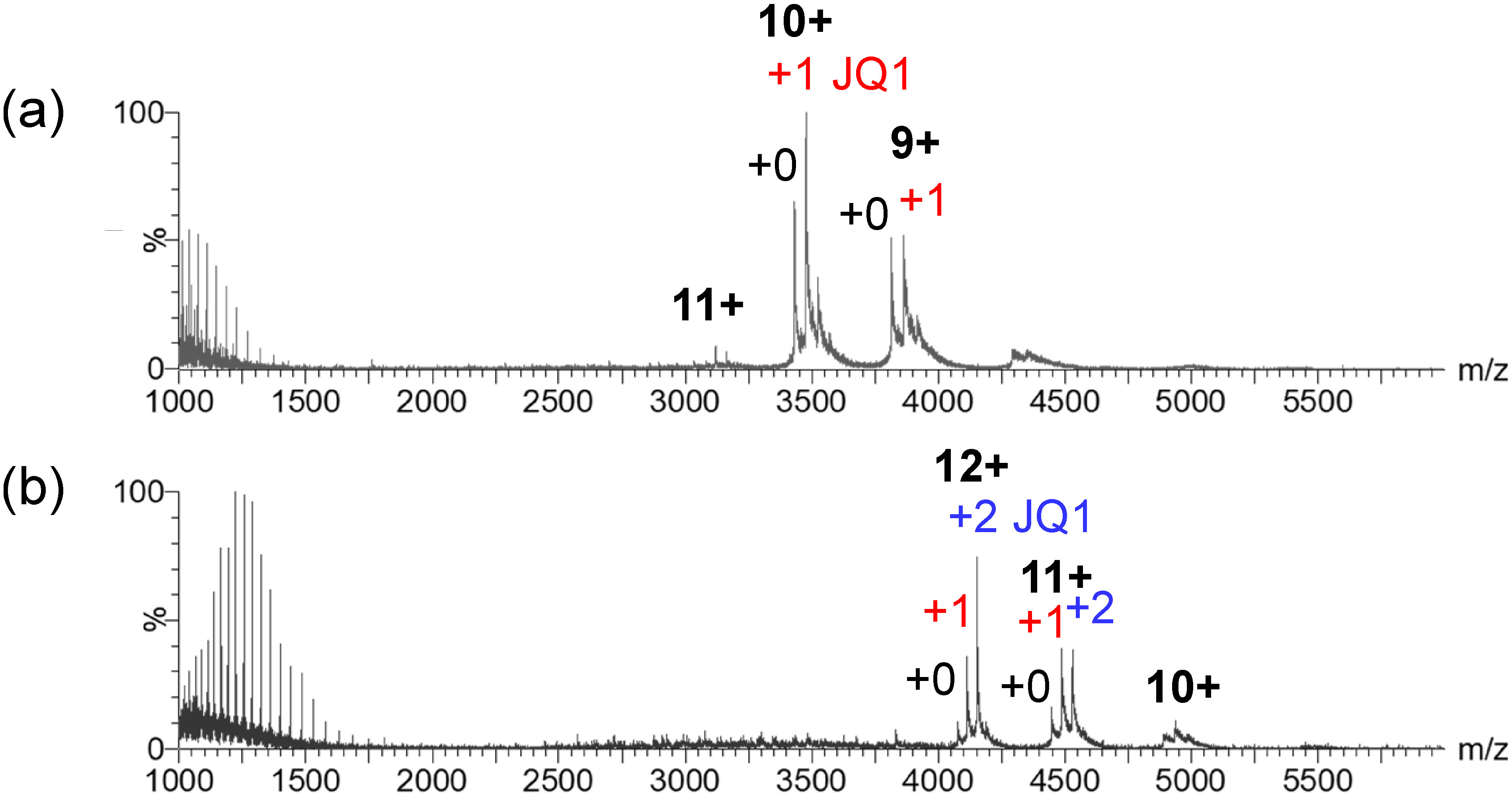
Fig. 6. NanoESI mass spectra of 5 μM (a) L-BD2 and (b) BD1-L-BD2 in the presence of 50 μM JQ1.

To observe protein–drug complexes, it is preferable to apply a minimum CE voltage to avoid the artificial dissociation of the drug in the mass spectrometer. However, considerable broadening of the protein peaks made it difficult to detect a clear mass shift by drug binding; thus, 20 V CE was applied in the analysis of the protein–JQ1 complexes for L-BD2 and BD1-L-BD2 (Fig. 6). For L-BD2 in the presence of 10 eq of JQ1 and 1% DMSO, we observed distinct peaks of the 1 : 1 complex. Complex formation was recognized only for 8+, 9+, 10+, and 11+ charged ions; all of the highly charged ions at *m*/*z* <1500 corresponded to JQ1-free L-BD2. A similar phenomenon was observed for BD1-L-BD2. As shown in Fig. 6, the latter displayed peaks for ions with 11+ and 12+ charges, which were bound to two, one, or zero JQ1 molecules. In contrast, the highly charged ions at *m*/*z* <1500 corresponded to JQ1-free BD1-L-BD2.

Samples were prepared in 100 mM ammonium acetate containing 1% DMSO. Spectra were obtained by applying 20 V of trap collision energy. The charge states are indicated in bold letters. The numbers of JQ1 molecules associated with the protein ions are indicated by black, red, and blue letters, respectively.

In the mass spectra of S-BD2 in the presence of JQ1, the S-BD2–JQ1 complex was nearly completely dissociated at 20 V CE. In contrast, the bromodomains in L-BD2 and BD1-L-BD2 retained JQ1, even at 20 V CE. This difference can be attributed to the fact that the increase in the internal energy of the high *m*/*z* ions upon collisions with Ar is smaller than that of the low *m*/*z* ions.^35)^

In the mass spectra of S-BD2 without a long IDR (in 100 mM ammonium acetate containing 1% DMSO), we observed ions at low-charge states that correspond to compact structures but ions at high-charge states that correspond to elongated conformers were not observed. In contrast, L-BD2 and BD1-L-BD2 (in 100 mM ammonium acetate containing 1% DMSO) exhibited bimodal charge distributions in the mass spectra, indicating the presence of both compact and elongated conformers. In the presence of JQ1, JQ1-bound proteins were observed only for the compact conformers of L-BD2 and BD1-L-BD2; no elongated conformers of the JQ1-bound complexes were found at *m*/*z* <2000. This suggests that the ions at *m*/*z* <2000 correspond to conformers with unfolded bromodomains. That is, S-BD2 without a long IDR retains the folded bromodomain, whereas some populations of L-BD2 and BD1-L-BD2 have unstructured bromodomains. Since the S-BD2 bromodomain alone presented only a compact conformer, this result suggests that the long disordered linker connected to it was responsible for destabilizing the bromodomain, leading to the generation of elongated conformers of L-BD2 and BD1-L-BD2, which cannot bind JQ1.

**Figure 7:**
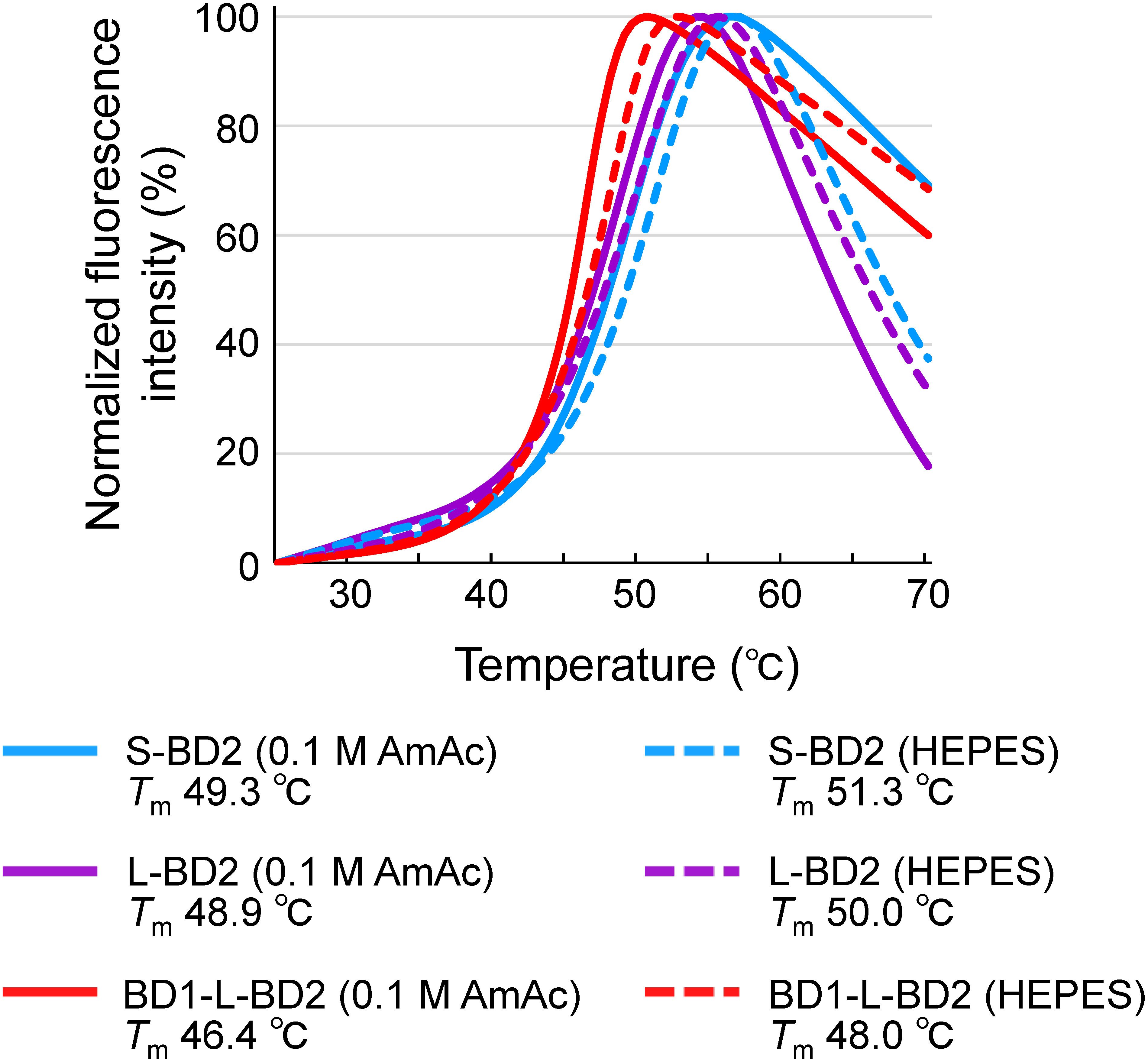
Fig. 7. Plots of the thermal stability analysis of S-BD2, L-BD2, and BD1-L-BD2.

To investigate the structural stability of the bromodomains in S-BD2, L-BD2, and BD1-L-BD2 in solution, a thermal stability assay, which enables protein denaturation to be monitored, was performed on the samples in 100 mM ammonium acetate or HEPES buffer (30 mM HEPES (pH 7.4) and 400 mM NaCl) (Fig. 7). By comparing their melting temperatures (*T*_m_), it was demonstrated that all proteins were more stable in HEPES buffer than in the ammonium acetate solution. This difference in structural stability could be due to differences in the pH or salt content of the solution. Furthermore, the S-BD2 bromodomain was the most stable among the three proteins in both solutions, with the overall structural stability among the bromodomains being S-BD2>L-BD2>BD1-L-BD2. It is likely that the linker region destabilized the folded domain, which is consistent with our native MS results.

The blue, purple, and red solid lines correspond to the plots for S-BD2, L-BD2, and BD1-L-BD2 in 100 mM ammonium acetate, respectively, and the blue, purple, and red broken lines correspond to the respective plots for S-BD2, L-BD2, and BD1-L-BD2 in 30 mM HEPES buffer (pH 7.4) and 400 mM NaCl. The *T*_m_ value of each sample is also shown.

In the case of BD1-L-BD2, the flexibility of the disordered linker region is slightly more limited than that of L-BD2. However, highly charged ions corresponding to unstructured conformers were found in the mass spectra for both L-BD2 and BD1-L-BD2. In a native MS analysis of the Swi5–Sfr1 complex, which contains a disordered region of ∼130 amino acid residues at the N-terminus of Sfr1, a relatively wide CCS distribution was observed without the dissociation of the complex.^16)^ In contrast, our present study suggests that some bromodomains in L-BD2 and BD1-L-BD2 were likely unfolded. The linker regions of L-BD2 and BD1-L-BD2 consisted of 221 and 201 residues, respectively, which are longer than the disordered region of the Swi5–Sfr1 complex. Thus, the length of the linker region may have affected the instability of the folded region in the gas phase. Furthermore, the linker region of BRD4 is rich in proline (Pro) and serine (Ser) with contents of 22.7% and 11.3%, respectively. The high Pro content in the linker region may have caused the low stability of L-BD2 and BD1-L-BD2. In any case, it is possible that the long disordered linker in the protein triggered the unfolding of the structured bromodomains in the gas phase.

## CONCLUSION

Native MS revealed that the bromodomain structure of BRD4 is retained in the majority of S-BD2, L-BD2, and BD1-L-BD2 proteins, but some bromodomain populations connected to the long disordered linker are unfolded and did not bind JQ1 in our study. Highly charged ions corresponding to elongated conformers were observed for BD1-L-BD2, which possesses a bromodomain at each end of the linker sequence, and the protein displayed a more restricted linker motility than L-BD2. For S-BD2, which has no long disordered linker, only low-charge ions were observed in the high-*m*/*z* region, implying a compact protein structure. Considering that the bromodomains of full length BRD4 recognize acetylated lysine residues in human cells, it is unreasonable to assume that some bromodomains connected to a long disordered linker cannot retain their folded structure. Comparing the properties of these proteins in the solution and gas phases, the destabilization of the bromodomain observed in some populations of L-BD2 and BD1-L-BD2 may be due to the pH of the ammonium acetate being slightly lower than that for physiological conditions or due to a specific phenomenon in the gas phase. In summary, we report on the successful characterization of proteins with BRD4 bromodomains and long disordered linkers using the charge-state distributions as observed in native mass spectra.

## Notes

Mass Spectrom (Tokyo) 2022; 11(1): A0110
