# Supplementary material for: Native Mass Spectrometry of BRD4 Bromodomains Linked to a Long Disordered Region
Source: Mass Spectrom (Tokyo). 2022 Dec 28;11(1):A0110. doi: 10.5702/massspectrometry.A0110 (PMC9853951; doi:10.5702/massspectrometry.A0110)
Supplement: Supplementary Data [file massspectrometry-11-1-A0110-s001.pdf]

Supplementary Materials

Native mass spectrometry of BRD4 bromodomains linked to a long-disordered region

Nanako Azegami[1], Rina Taguchi[1], Noa Suzuki[2], Yusuke Sakata[1], Tsuyoshi Konuma[1,2], Satoko Akashi[1,2]

[1] Graduate School of Medical Life Science, [2] School of Science,
Yokohama City University, 1-7-29 Suehiro-cho, Tsurumi-ku, Yokohama, Kanagawa 230-0045, Japan

S-BD2

```
GSSKVSEQLK CCSGILKEMF AKKHAAYAWP FYKPVDVEAL
GLHDYCDIIK HPMDMSTIKS KLEAREYRDA QEFGADVRLM
FSNCYKYNPP DHEVVAMARK LQDVFEMRFA KMPDE
```

L-BD2

```
SMEETEIMIV QAKGRGRGRK ETGTAKPGVS TVPNTTQAST
PPQTQTPQPN PPPVQATPHP FPAVTPDLIV QTPVMTVVPP
QPLQTPPPVP PQPQPPPAPA PQPVQSHPPI IAATPQPVKT
KKGVKRKADT TTPTTIDPIH EPPSLPPEPK TTKLGQRRES
SRPVKPPKKD VPDSQQHPAP EKSSKVSEQL KCCSGILKEM
FAKKHAAYAW PFYKPVDVEA LGLHDYCDII KHPMDMSTIK
SKLEAREYRD AQEFGADVRL MFSNCYKYNP PDHEVVAMAR
KLQDVFEMRF AKMPDEPEEP VVAVSSPAVP PPT
```

BD1-L-BD2

```
SMNPPPPETS NPNKPKRQTN QLQYLLRVVL KTLWKHQFAW
PFQQPVDAVK LNLPDYYKII KTPMDMGTIK KRLENNYYWN
AQECIQDFNT MFTNCYIYNK PGDDIVLMAE ALEKLFLQKI
NELPTEETEI MIVQAKGRGR GRKETGTAKP GVSTVPNTTQ
ASTPPQTQTP QPNPPPVQAT PHPFPAVTPD LIVQTPVMTV
VPPQPLQTPP PVPPQPQPPP APAPQPVQSH PPIIAATPQP
VKTKKGVKRK ADTTTPTTID PIHEPPSLPP EPKTTKLGQR
RESSRPVKPP KKDVPDSQQH PAPEKSSKVS EQLKCCSGIL
KEMFAKKHAA YAWPFYKPVD VEALGLHDYC DIIKHPMDMS
TIKSKLEARE YRDAQEFGAD VRLMFSNCYK YNPPDHEVVA
MARKLQDVFE MRFAKMPDEP EEPVVAVSSP AVPPPT
```

Figure S1. Amino acid sequences of S-BD2 (347-460), L-BD2 (167-477), and BD1-L-BD2 (44-477).

Blue and red letters correspond to structured BD1 and BD2 bromodomains, respectively. Underlined sequences correspond to linker regions to the affinity tags that were remained after the removal of the tags by protease cleavages.

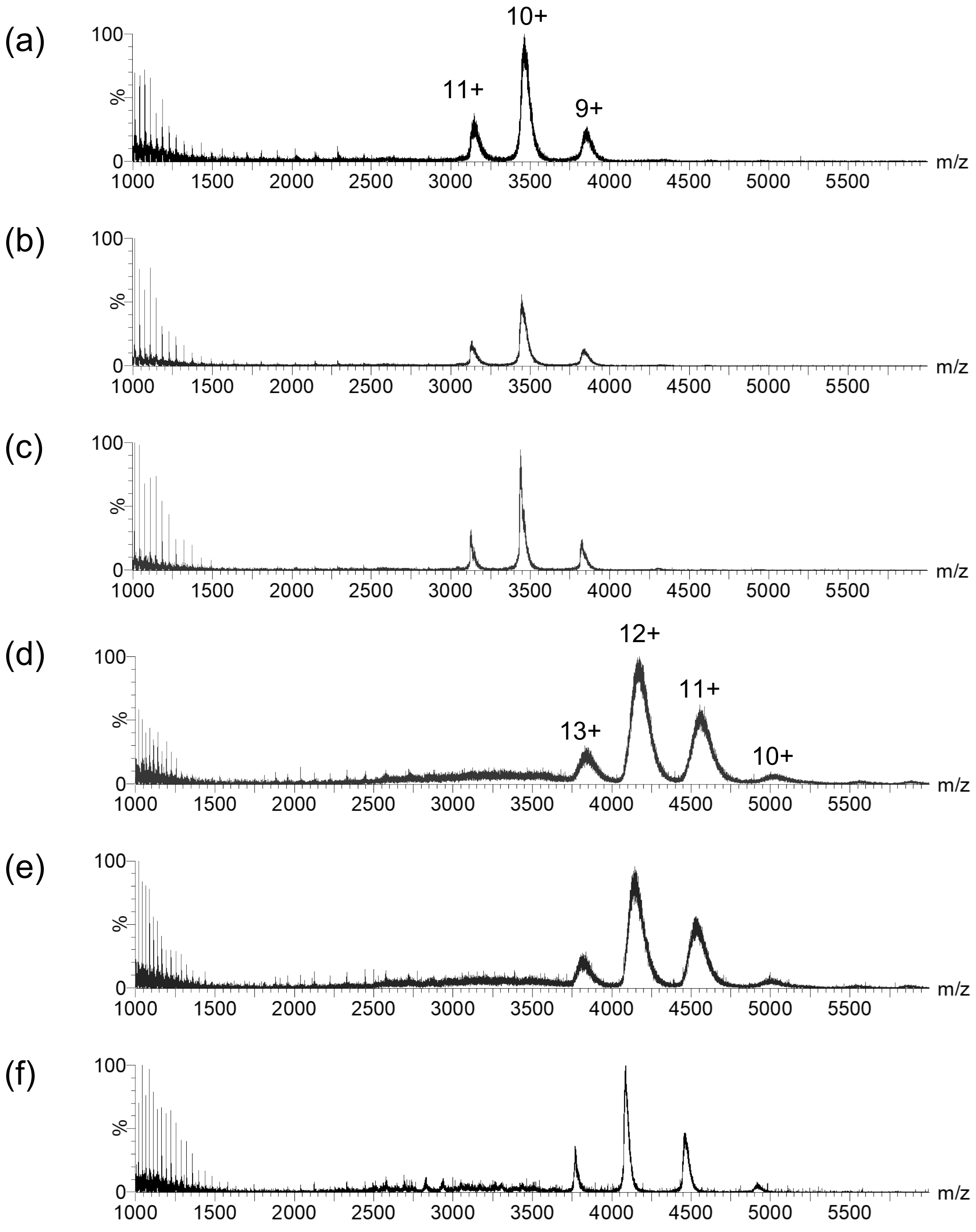

Figure S2. NanoESI mass spectra of 5 μM (a–c) L-BD2 and (d–f) BD1-L-BD2 by applying 4 V (for (a), (d)), 10 V (for (b), (e)), and 15 V (for (c), (f)) of trap collision energy. Samples were prepared in 100 mM ammonium acetate containing 1% DMSO.

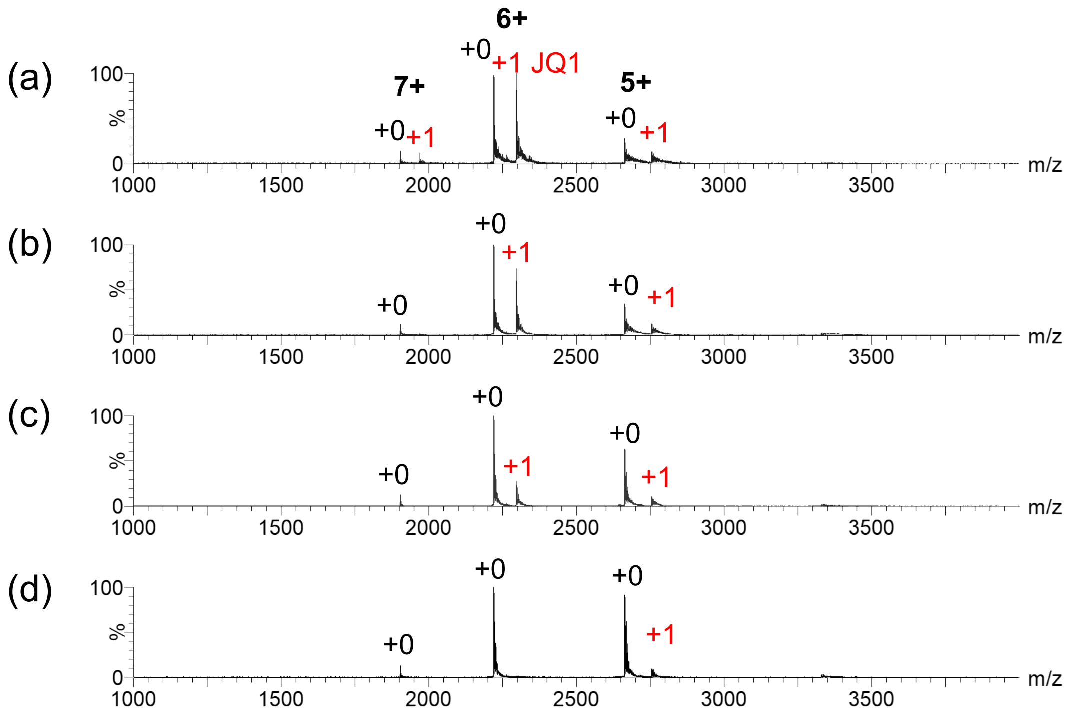

Figure S3. NanoESI mass spectra of 5 μM S-BD2 in the presence of 5 μM JQ1 by applying (a) 4 V, (b) 10 V, (c) 15 V, and (d) 20 V of trap collision energy.

Samples were prepared in 100 mM ammonium acetate containing 1% DMSO. The charge states are indicated in bold letters. The numbers of JQ1 molecules associated with the protein ions are indicated by black and red letters.
